# Attitudes vs. Purchase Behaviors as Experienced Dissonance: The Roles of Knowledge and Consumer Orientations in Organic Market

**DOI:** 10.3389/fpsyg.2017.00248

**Published:** 2017-02-24

**Authors:** María Hidalgo-Baz, Mercedes Martos-Partal, Óscar González-Benito

**Affiliations:** ^1^Department of Business Economics and Administration, University of SalamancaSalamanca, Spain; ^2^IME Instituto Multidisciplinar de Empresa (Multidisciplinary Institute for Enterprise), University of SalamancaSalamanca, Spain

**Keywords:** organic products, environmental protection orientation, health orientation, hedonic orientation, organic market knowledge, cognitive dissonance theory

## Abstract

This research focuses on the incongruity between positive attitudinal responses but a lack of purchase behavior in organic markets. According to cognitive dissonance theory, consumer orientations toward the benefits attributed to organic products (environmental protection, health, hedonic) relieve the dissonance that results from this attitude–behavior incongruity. Knowledge also functions as a transmitter, from positive attitudes to purchase behaviors, thereby reducing the incongruity. Using quota sampling in a survey study, this paper tests the hypotheses from linear regression models. The results show that orientations and knowledge improve the congruity between attitudes and purchase behaviors toward organic products. Moreover, interaction effects arise between the environmental protection orientation and knowledge and between the hedonic orientation and knowledge. Increasing knowledge mitigates the difference between attitudes and purchase behaviors, especially for consumers with environmental protection or hedonic orientations. These findings have several important implications for research and practice.

## Introduction

Health concerns and environmental protection are increasingly important societal issues (Bhattacharya and Sen, 2004; Moisander, 2007; Groening et al., 2009; Pagiaslis and Krystallis-Krontalis, 2014; Nielsen, 2015), leading to the developments of green and organic markets. Whereas green markets focus on social and environmental responsibilities (Akehurst et al., 2012), organic markets address broader consumer concerns for health, environmental protection, and food safety by relying on agricultural systems that are free of human-made chemicals (Pino et al., 2012). Although related, these markets differ, in that the organic market entails not just environmental issues but also health and food safety concerns. With its focus on health and environmental protection (Zanoli and Naspetti, 2002; Baker et al., 2004; Essoussi and Zahaf, 2008; Bauer et al., 2013), the organic market also has experienced huge growth (Kareklas et al., 2014), transforming from a niche to a central product trend in the food industry (Van Doorn and Verhoef, 2015). Global sales of organic food and beverages reached US$72 billion in 2013 (Willer and Lernoud, 2015), a nearly 5-fold increase over 1999 sales (US$15.2 billion), and then expanded even further to US$80 billion in 2014 (IFOAM – Organics International, 2016). In Spain, expenditures on organic products reached 1018 million euros in 2013, equivalent to a 5.5% increase in sales compared with 2011 (Prodescon, 2014).

Parallel with this growing social interest and rapid sales expansions, academic research into the organic market has increased as well (Kim and Chung, 2011). Hughner et al. (2007) classify consumer responses to organic products, according to their consideration in previous literature. For example, some studies address factors that facilitate or encourage these responses, whereas others focus on inhibiting factors. Among the former, most studies concentrate on motivations or consumer orientations related to environmental protection and health (Borin et al., 2011; Kim and Chung, 2011; Bauer et al., 2013), as well as prosocial or altruistic values, together with hedonic or self-benefiting values (Cornelissen et al., 2008; Urien and Kilbourne, 2011; Yang et al., 2015). Among the latter, negative factors, we find studies of price, consumer confidence (Bhaskaran et al., 2006; Terrachoice Environmental Marketing, 2009; Gleim et al., 2013), and ineffective marketing (Krystallis et al., 2006; Hughner et al., 2007; Aertsens et al., 2009; Ngobo, 2011). Such factors all can influence consumers' attitudes toward and purchases of organic products (Tsakiridou et al., 2008; Ngobo, 2011; Akehurst et al., 2012; Bickart and Ruth, 2012; Tucker et al., 2012; Atkinson and Rosenthal, 2014).

However, attitudes toward organic products appear to differ from purchase behavior in this market. That is, sustainable consumption and healthy eating remain top priorities among modern consumers (Pagiaslis and Krystallis-Krontalis, 2014; Nielsen, 2015), yet an attitude–behavior gap or values–action gap arises, such that consumer express environmental concerns, but those concerns do not translate into purchase behaviors (Akehurst et al., 2012). Therefore, understanding the behaviors of organic consumers, the antecedents of organic consumption, and the incongruity between attitudes and behavior is critical. According to Akehurst et al. (2012), who study the difference between green purchase intentions and green purchase behaviors, this gap is less evident for consumers with high ecological consciousness.

The current research in turn investigates the incongruity between consumer responses, in the form of attitudes, and their behaviors, in the form of purchases, of organic products. We focus on the organic food market instead of the green market. Using cognitive dissonance theory (Festinger, 1957), we study the incongruity by analyzing the role of consumer orientation in relation to consumer behavior (Kim and Chung, 2011; Akehurst et al., 2012; Izagirre-Olaizola et al., 2013; Van Doorn and Verhoef, 2015). Specifically, we consider the potential effects of environmental protection, health, and hedonic (i.e., taste and satiety) orientations, together with consumer knowledge about organic products.

In the next section, we review previous literature and develop our hypotheses. After detailing our methodology and analysis, we present the study results. Finally, we discuss some conclusions, implications, and limitations of this study, as well as ideas for further research.

## Theoretical framework

### Effect of knowledge on attitude–behavior incongruity in organic markets

Even when consumers state very positive attitudes toward organic or green products, they frequently exhibit incongruous behaviors and fail to purchase these products. That is, a positive attitude does not translate into a purchase (D'Souza et al., 2007; Pickett-Baker and Ozaki, 2008; Florenthal and Arling, 2011; Moraes et al., 2012; Gleim et al., 2013). Thus, organic market is characterized by an attitude-behavior incongruity. Ongoing studies seek to explain this incongruity, using a variety of factors. For example, organic food consumption might be barred by high prices, lack of consumer confidence (Bhaskaran et al., 2006; Terrachoice Environmental Marketing, 2009; Gleim et al., 2013), or lack of consumer knowledge about these products. A lack of knowledge makes it difficult for consumers to differentiate organic from conventional products (Chryssochoidis, 2000; Gleim et al., 2013; Gfk, 2014). Thus, increased knowledge might be a key factor that would encourage organic purchase behavior. Aertsens et al. (2009) also note that providing more information or increasing awareness of organic products can help lower consumers' uncertainty about the unique attributes of organic offerings, as well as mitigate their lack of confidence about certification methods. Such reduced uncertainty then might improve purchase likelihood (Thøgersen, 2007).

Overall, if more knowledge about organic products influences consumers' decisions and increases their willingness to pay (Barnes et al., 2009), it should have a positive effect on organic product purchases, while also attenuating other consumption barriers, such as a lack of confidence and high prices (Aertsens et al., 2009; Barnes et al., 2009). Knowledge thus might function as a transmitter, from attitudes to purchase behaviors. Therefore,

H1. More knowledge about organic products leads to greater congruity between consumers' attitudes and purchase behaviors.

### Cognitive dissonance

According to cognitive dissonance theory, each person maintains a cognitive view of him- or herself, past behaviors, beliefs, attitudes, and environments (Oshikawa, 1968). Elements of this view might become dissonant if they are inconsistent or contradict each other. In an organic market setting for example, consumers express positive attitudes toward organic products but do not buy them, so they might experience dissonance between their own attitudes and behaviors. Therefore, we define the incongruity characterizing organic market as a dissonance arised from contradictory responses that consumers state. Cognitive dissonance theory suggests that such inconsistencies generate a disturbing, unpleasant sensation for the consumer, who then tries to avoid or prevent the inconsistency (Festinger, 1957).

Dickerson et al. (1992) argue that this sensation of dissonance can result from hypocrisy, due to a discrepancy between actual behaviors and norms for what people should do to benefit the environment, according to their own beliefs, concerns, or orientations. Nonetheless, when faced with an incongruity between their attitudes and purchase behaviors in the organic market, consumers likely seek to modify the dissonant elements (Oshikawa, 1968), in accordance with their concerns or orientations. In fact, Cornelissen et al. (2008) point out that previous behavior of a consumer is used as a heuristic basis for later decisions. Therefore, consumer may change their purchase behavior toward organic products, rather than their attitudes. That way, consumers may show different orientations in their current behavior, based on their perception about their previous behavior. In this respect, Becker et al. (1977) show a health orientation leads people to engage in healthy behaviors; and Schlegelmilch et al. (1996) show an environmental orientation prompts them to make green decisions.

More broadly, orientations related to the attributes and benefits of organic products should lead consumers to relax or correct the cognitive dissonance they experience, due to the difference between their attitudes and purchase behaviors, by increasing their purchase responses. In organic markets, the benefits associated with the products are mainly environmental protection and health (Essoussi and Zahaf, 2008; Kareklas et al., 2014). Relative to conventional products, organic products generally are perceived as offering more nutritional value and being produced in a more natural way, without chemicals or harmful pesticides (Ott, 1990; Wilkins and Hillers, 1994; Wandel and Bugge, 1997; Squires et al., 2001; Pino et al., 2012). In this sense, organic products also are assumed to be more environmentally friendly (Wilkins and Hillers, 1994; Hughner et al., 2007).

Consumer orientations related to these organic benefits (i.e., environmental protection and health) therefore should have a positive impact on the purchases of organic products, thereby reducing the difference between attitudes and purchase behaviors. Akehurst et al. (2012) concur that the gap between purchase intentions and purchases is less evident for green products when consumers' environmental consciousness is high. Thus, we propose:

H2a. Consumers' higher environmental orientation leads to greater congruity between their attitudes and purchase behaviors toward organic products.
H2b. Consumers' higher health orientation leads to greater congruity between their attitudes and purchase behaviors toward organic products.

We also consider hedonic orientations, because previous research indicates that consumers perceive organic products as tastier and offering better visual appearances and scent (McEachern and McClean, 2002; Cervellon and Carey, 2014). For example, McEachern and McClean (2002) link perceptions of better flavor to the increased safety associated with organic food and cite these notions as the primary reasons consumers buy organic products. Cervellon and Carey (2014) also note that consumers consider the hedonic attributes of organic food, such as their visual appearance, scent, and texture, more positively in their post-purchase assessments. Therefore, consumers with a more hedonic orientation might be more consistent in their attitudes and purchase behaviors toward organic products, such that they may experience less dissonance. In further support of this prediction, Van Doorn and Verhoef (2015) show that consumers oriented toward product quality and taste are less concerned about prices. Therefore, the negative effect of the price premium on the purchase of organic food may be weaker for consumers concerned about the quality and taste of the food. This preference and orientation can help overcome the barriers of organic consumption and facilitate the translation of positive attitudes into purchases. Therefore,

H2c. Consumers' higher hedonic orientation leads to greater congruity between their attitudes and purchase behaviors toward organic products.

### Moderating effect of knowledge

Previous literature has analyzed environmental concerns and knowledge about organic products as factors that might explain organic or green purchase behavior (Kollmuss and Agyeman, 2002; Mostafa, 2007; Pagiaslis and Krystallis-Krontalis, 2014). For example, Pagiaslis and Krystallis-Krontalis (2014) propose a mediation relationship, following a sequence of orientation–knowledge and belief–behavior, such that consumers who are more oriented toward environmental protection are also more informed and have more positive beliefs about green products. Therefore, knowledge and beliefs may be necessary for purchases of these products to take place.

We propose that knowledge might be a moderator, as well as a mediator, in these relationships. Beyond organic literature, knowledge is considered as moderator in the relationship attitude-behavior. Specifically, Berger et al. (1994) take into account that moderating variable in the relationship between attitude and ecological behavior by studying different kinds of heating systems. Those authors note knowledge increases the attitude strength and, consequently, the effect of the attitude on behavior will be greater. In fact, subjective knowledge will be an important indicator in high involvement, high risk, search product categories. Information is collected over time for those cases. So, attitude would be stronger as subjective knowledge increases and, accordingly, its effect on behavior.

We have predicted that the dissonance experienced as a result of incongruity between attitudes and behaviors might be lower among consumers with more knowledge, such that knowledge might overcome some of the barriers to the consumption of organic products. In this sense, it could facilitate the transformation from positive attitudes to purchases of organic food. Nonetheless, we propose knowledge as moderator in the relationship between orientations and attitude-behavior congruence as well. We expect that more informed consumers, who know the attributes and benefits of organic products, respond in ways that are more consistent if their orientations also are linked to these attributes and benefits. Consumers with more knowledge about organic food will buy even more if their orientations also are aligned with the benefits attributed to organic products, such that the difference between their attitudes toward organic food and their purchase behavior will be smaller. This reasoning implies interaction effects between knowledge and orientations, such that knowledge accentuates the positive effect of an environmental orientation, health orientation, or hedonic orientation on the congruence between attitudes and purchase behaviors toward organic food. In other words, knowledge positively moderates the relationship between consumers' orientations and the congruence between their attitudes and behavioral responses.

H3a. Consumers' environmental orientation exerts a stronger effect on the congruence between their attitudes and purchase behaviors when consumers have more knowledge of organic products.
H3b. Consumers' health orientation exerts a stronger effect on the congruence between their attitudes and purchase behaviors when consumers have more knowledge of organic products.
H3c. Consumers' hedonic orientation exerts a stronger effect on the congruence between their attitudes and purchase behaviors when consumers have more knowledge of organic products.

## Methods

To test the hypotheses and obtain pertinent empirical evidence, we conducted a survey in an urban area in Castilla y Leon, Spain. A pretest prior to the main data collection ensured the comprehensibility of the items within the survey, as well as the appropriateness of the data collection procedure. Respondents reported to be responsible for or actively involved in purchasing food for their households. We ensured that the data came from a wide range of ages and both genders, using a quota sampling method. Finally, after studying the existence of outliers, we use data from 305 (out of 311 obtained initially) consumers that have been collected between April and June 2013.

The information requested in the survey refers to the responses of consumers to organic food, the benefits sought, consumer orientations and their values, and socio-demographic characteristics. A seven-point Likert scale (0 = “strongly disagree” and 6 = “strongly agree”) applied to all the items except for sociodemographic characteristics.

### Responses to the organic product

We consider three consumer responses: cognitive, affective, and conative. A cognitive response implies awareness of the existence of the object, retained information, and knowledge about an object. An affective response refers to the emotions a person feels relative to objects or events, such as a preference or dislike of a product or service. Finally, conative response implies the form of the reaction, such as a purchase (Lavidge and Steiner, 1961; Lambin, 1993; Peter and Olson, 2005). All consumer responses are built as means and scale reliability is assessed from Pearson Correlation and Cronbach's Alpha, as Table 1 shows. Those coefficients confirm the suitability of our measures about consumer responses. Additionally, we check construct validity using Confirmatory Factor Analysis (CFA) that shows a good model fit (χ^2^ statistic: 10.608 (0.101); CFI: 0.994; RMSEA: 0.050).

**Table 1:** **Consumer responses to organic food**.

**Variables**	**Items**	**M**	***SD***	**PC**	**α Cronbach**
Knowledge	I know the benefits and attributes of organic products	2.160	1.414	0.508^**^	0.667
	I know how to differentiate organic products from conventional products.				
Attitude	I would prefer to buy an organic product rather than a conventional one at the same price	4.872	1.269	0.847^**^	0.917
	Buying organic products is a good choice for me.				
Purchase	I usually buy organic products	1.325	1.347	0.647^**^	0.783
	It would be difficult for me to dispense with organic products in my shopping cart.				
Purchase Deducting Attitude (PDA)		−3.548	1.437	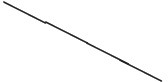	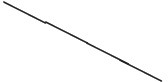

M, mean; SD, standard deviation; PC, pearson correlation/

** *p < 0.01*.

The knowledge variable measures cognitive responses. We use items adapted from scale of Pagiaslis and Krystallis-Krontalis (2014) about subjective knowledge. So, our variable is constructed as the average of two items that refer to consumer knowledge about the attributes of organic products and the differentiation from their counterpart, conventional products.

Attitude toward organic foods constitutes the measure of affective responses; it is constructed as the average of two items pertaining to overall assessments of organic products and consumer preferences. This measure is congruent with the attitude used by Ajzen and Madden (1986). Those authors define specifically the attitude as an overall assessment related to different consumer beliefs about a certain object or product.

We also calculate the average of two items to measure the conative response, as reflected in the purchase variable. Likewise, our measure is congruent with the measure proposed by Ajzen and Madden (1986) dealing with the likelihood of certain behavior. In this case, the two items pertain to the consumer's purchase and intention to consume organic products.

The “purchase deducting attitude” (PDA) variable is the difference resulting when we subtract the attitude variable from the purchase variable. Thus, it reflects the level of congruence between purchase behaviors and attitudes. In our sample, all respondents indicated more positive attitudes than purchase behaviors, such that the PDA values range from −6 to 0. Consumers with a positive attitude who buy organic products frequently (i.e., exhibit congruent responses) take values near 0. But if consumers indicate more incongruent responses, the PDA values are more negative and farther away from 0.

### Consumer orientations

We used six items adapted from the “Self-perception” scale of Cornelissen et al. (2008) to measure consumer orientations. Hence, we consider orientations as self-perception of the consumer about their past behavior related to the different benefits of organic product. Specifically, we extracted three orientations: environmental orientation, health orientation, and hedonic orientation. Every orientation is calculated as the average of two items about self-perception of consumer related to either proenvironmental behavior or health behavior or hedonic behavior, depending on the orientation. Therefore, those measures are assessed similarly to consumer responses, scale reliability is measure with Pearson Correlation and Cronbach's Alpha. The statistical results for this assessment are in Table 2. Although those Cronbach's Alphas are lower than the common rule of 0.7 some update research point out that “there is no universal minimally acceptable reliability value. An acceptable reliability value depends on the type of application, and furthermore, the focus should be on the population reliability value and not on the sample reliability value” (Bonett and Wright, 2015, p. 4). Similarly to consumer responses, we check construct validity using CFA and the model fit is good (χ^2^ statistic: 7.367 (0.288); CFI: 0.994; RMSEA: 0.027).

**Table 2:** **Consumer orientations to organic food**.

**Variables**	**Items**	**M**	***SD***	**PC**	**α Cronbach**
Environmental Orientation	I think my behavior is eco friendly.	3.084	1.304	0.441^**^	0.594
	I take into account the environmental impact when I buy food.				
Health Orientation	I think my behavior is responsible for my health.	4.298	1.144	0.383^**^	0.554
	I take into account the impact on my health and fitness when I buy food.				
Hedonic Orientation	I usually indulge in eating some kinds of food.	3.443	1.500	0.425^**^	0.585
	Some food intake makes me feel better, happier.				

M, mean; SD, standard deviation; PC, pearson correlation/

** *p < 0.01*.

### Consumer characteristics

The survey also gathered demographic profiles, reflecting the respondents' gender, age, household size, and presence of children (younger than 6 years or 7–12 years old) in the house. Gender was a dichotomous variable (0 = male, 1 = female). Women represent 86.5% of the sample. Age is a count variable; 45.85 years is the average (*SD* = 12.06). Open questions assess both household size and the number of children. Household size is a count variable, with the following distribution: one-member households (19.6%), two members (27.7%), three members (28.3%), four members (21.2%), and five or more members (3.1%). Finally, 10.6% of respondents have children younger than 6 years, and 11.6% have children between 7 and 12 years of age in their households.

## Results

We use linear regression models to test the hypotheses. The results and their interpretations reflect widely accepted significance values (*p* < 0.05 and *p* < 0.01), though in some cases, we consider values of *p* < 0.10 as well. The following specification provides the test for our first hypothesis and second block of hypothesis:

(1) $$\begin{array}{r} \text{PDA}=\alpha +\Sigma \beta \left(\text{CV}\right)+\gamma \text{Knowledge}+\Sigma \sigma \left(\text{O}\right)+\xi , \end{array}$$

where PDA is the “purchase deducting attitude” variable, α is a constant we use to estimate the model, CV is the vector of the control variables in the study (gender, age, household size, number of children), β denotes the vector of parameters to estimate the effect of the control variables, γ is a parameter to estimate the effect of knowledge, O represents the vector of variables related to consumer orientations (environmental, health, and hedonic), σ denotes a vector of parameters we use to estimate the effect of the variables related to those consumer orientations, and ξ refers to the error term of the model.

The estimation results of our tests of H1 and H2a–c are in the first column of the Table 3. The effect of knowledge on PDA is positive and significant at a confidence level greater than 95%, in support of H1. Higher knowledge leads to greater congruity in consumer responses toward organic products; consumers who are more familiar with organic products buy more of them. In turn, the difference between their attitudes and purchase behaviors shrinks. According to the coefficients in Table 3, an environmental orientation has a positive effect on PDA too (95% confidence level). For the health orientation and hedonic orientation, there is no significative effect. Nevertheless, they are congruent with our hypothesis. Although no signiticative, their effects are positive. In conclusion, these results offer only support for H2a. Consumers oriented toward the environment, which also is the main benefit gained from organic food, have more consistent responses to organic products.

**Table 3:** **Effects of knowledge and consumer orientations on congruence in consumer responses to organic food**.

	**PDA (1)**	**PDA (2)**
**CONTROL VARIABLES**		
Constant	−5.573^**^	−5.494^**^
Gender (1 = female)	−0.222	−0.071
Age	0.016^*^	0.014^+^
Household size	−0.005	0.013
Children under 6 years old	0.121	0.110
Children from 7 to 12 years old	−0.315^+^	−0.294
**DIRECT EFFECTS**		
Knowledge	0.142^*^	0.073
Environmental orientation	0.166^*^	−0.159
Health orientation	0.101	0.350^*^
Hedonic orientation	0.077	0.013
**INTERACTIONS**		
Knowledge x Environmental orientation	−	0.134^**^
Knowledge x Health orientation	−	−0.104^+^
Knowledge x Hedonic orientation	−	0.018
**COEFFICIENT OF DETERMINATION (R^2^)**	0.103^**^	0.139^**^

+ *p < 0.10*,

* *p < 0.05*,

** *p < 0.01*.

To test H3a–c, we use the following specification:

(2) $$\begin{array}{l} \text{PDA}=\alpha +\Sigma \beta \left(\text{VC}\right)+\gamma \text{Knowledge}+\Sigma \sigma \left(\text{O}\right)\\ +\Sigma \lambda \left(\text{Knowledge }\times \text{ O}\right)+\xi , \end{array}$$

where (Knowledge × O) denotes the vector of variables reflecting the interactions between knowledge and consumer orientation, and λ is the vector of parameters we use to estimate the effect of these interactions.

The estimation results for H3 are in the second column of the Table 3. Firstly, we check changes for coefficient of determination (*R*^2^) between a regression model about direct effects only (first column of Table 3) and a regression model including interaction effects (second column of Table 3). Comparing their R^2^, it proves that interaction effects get to improve the goodness of model fit for the two presented regressions on the table. As Table 3 shows, both the knowledge–environmental orientation and the knowledge–health orientation interactions are significant (95 and 90% confidence levels, respectively). But, those results show only support for knowledge–environmental orientation interaction (H3a). They do not offer support for H3b: the interaction between knowledge and health orientation is negative, opposite of our predictions. Hughner et al. (2007) note that most research identifies health as the main reason for buying organic food, but other authors, such as Williams (2002), find no conclusive evidence of an effect of organic food on people's health, compared with conventional food, which might explain this opposite result. That is, increasing knowledge does not reduce the incongruence between attitude and purchase behavior for health-oriented consumers. Instead, more knowledge of organic products strengthens the relationship only for environmental orientation in terms of the congruity in their attitudes and purchase behaviors. In brief, only consumers with environmental orientation will express more congruent attitudes and purchase behaviors when they know more about organic food, but consumers with a health orientation or hedonic orientation do not.

To check the heteroscedasticity of all estimated models, we ran a Breusch-Pagan test, one of the most common tests of heteroscedasticity. This test rejects homoscedasticity in the two models we used to test our hypothesis. Thus, we used robust estimations for the regression models appearing in the Table 3.

## Discussion

The empirical results confirm both that knowledge and environmental orientation of consumers influence the congruity between their attitudes and purchase behavior when it comes to organic food. We also find significant interaction effects across these factors, such that more knowledgeable people with environmental orientation have more congruent responses, and their attitudes and purchase behaviors toward organic products in turn are more similar.

### Theoretical implications

In line with previous literature that suggests incongruities between attitudes and purchase behaviors toward environmental or green products (Kollmuss and Agyeman, 2002; Akehurst et al., 2012; Moraes et al., 2012), we find that consumers have very positive attitudes toward organic products, with a mean of 4.9 on a 0–6 scale, whereas their purchase behaviors are incongruent, with a mean of 1.3 on the same scale (see Table 1). This incongruity likely reflects the predictions of cognitive dissonance theory. This theory predicts that people can experience dissonance over a wide range of dimensions (e.g., behaviors, attitudes, beliefs), such that the set of possible mechanisms to mitigate the dissonance also is broad. Our results suggest that consumer orientations reflecting the environmental benefits of organic products, and consumers' knowledge about organic products both are mechanisms that can reduce incongruities between attitude and purchase behaviors in the organic market.

Consistent with Akehurst et al. (2012), who find a gap between purchase intentions and purchase behaviors for green products, which diminishes among people with greater ecological consciousness, we find a difference between attitudes and purchase behaviors. The experienced dissonance in turn can be explained by consumer orientations related to environment. Consumers with an environmental-protection orientation exhibit more similar responses, such that the gap between their attitudes and their purchase behaviors is smaller.

Knowledge also helps explain the dissonance resulting from this incongruity. This type of response is not only a mediator for the relationship between attitude, as a measure of overall belief about the product, and organic purchase as Pagiaslis and Krystallis-Krontalis (2014) propose for their relationship beliefs-behavioral intention. It is also a moderator. Knowledge helps transmit attitudes to purchase behaviors, overcoming several barriers to organic consumption, such as a lack of consumer confidence and high prices. Knowledge also moderates the effect of environmental orientation on the congruence between attitudes and purchases of organic food. For consumers with more information about the environmental benefits of organic products, the relationship between that orientation and the congruence between attitudes and purchase behaviors is stronger.

### Limitations and further research

Some limitations of this study suggest directions for further research. We use only two items per factor about orientations. The results related to low Cronbach's Alpha of orientations could get better, for example, adding more items to measure these complex factors. On the other hand, we focus on factors that facilitate consumption and inhibit incongruity; we ignore factors that might inhibit consumption and facilitate incongruity, such as consumers' willingness to pay. Furthermore, we use consumers' assessments of their own purchases, rather than actually observed organic purchase data. This measure could lead to an underestimate of the actual level of incongruity between their attitudes and purchase behaviors. Other variables, such as social influence or social pressure, also could affect consumers' purchases of organic foods and their attitudes, such that they might increase or decrease the gap. Social influence also might be direct, or it could moderate the effect of the consumer orientations on congruity between attitudes and purchase behaviors. Thus, additional research thus should consider factors that inhibit purchases, along with other variables that were not included herein, such as the effect of social pressure on organic purchase behaviors, to analyze the incongruity between attitudes and purchase behaviors in this market.

## Author contributions

All authors listed, have made substantial, direct and intellectual contribution to the work, and approved it for publication.

## Funding

This research was supported by Ministerio de Educación y Ciencia, Grant ECO2014-53060-R (Spain).

### Conflict of interest statement

The authors declare that the research was conducted in the absence of any commercial or financial relationships that could be construed as a potential conflict of interest.
